# Plasmodial sugar transporters as anti-malarial drug targets and comparisons with other protozoa

**DOI:** 10.1186/1475-2875-10-165

**Published:** 2011-06-15

**Authors:** Ksenija Slavic, Sanjeev Krishna, Elvira T Derbyshire, Henry M Staines

**Affiliations:** 1 Centre for Infection, Division of Cellular and Molecular Medicine, St. George's, University of London, Cranmer Terrace, London SW17 0RE, UK; 2 Centre for Parasitic Zoonoses, Institute for Medical Research, University of Belgrade, Dr. Subotica 4, 11129 Belgrade, Serbia

## Abstract

Glucose is the primary source of energy and a key substrate for most cells. Inhibition of cellular glucose uptake (the first step in its utilization) has, therefore, received attention as a potential therapeutic strategy to treat various unrelated diseases including malaria and cancers. For malaria, blood forms of parasites rely almost entirely on glycolysis for energy production and, without energy stores, they are dependent on the constant uptake of glucose. *Plasmodium falciparum *is the most dangerous human malarial parasite and its hexose transporter has been identified as being the major glucose transporter. In this review, recent progress regarding the validation and development of the *P. falciparum *hexose transporter as a drug target is described, highlighting the importance of robust target validation through both chemical and genetic methods. Therapeutic targeting potential of hexose transporters of other protozoan pathogens is also reviewed and discussed.

## Background - Malaria burden and drug resistance

Today drug-resistant malaria is a persistent global health threat, resulting in an estimated one million human deaths worldwide. Of all malarial species, infection with *Plasmodium falciparum *is the cause of the greatest death toll, hitting sub-Saharan Africa hardest. Following the emergence of chloroquine resistance more than half a century ago, new drugs were introduced as alternative treatment regimens. The efficacy of these drugs deteriorated quickly, for some of them at an alarming rate, as malarial parasites evolved multiple mechanisms of drug resistance. For example, the first reports of sulphadoxine-pyrimethamine and atovaquone resistance arrived in the same year as their introduction [1]. With worsening resistance to all available anti-malarials in Southeast Asia, artemisinins, extracted from a plant used in traditional Chinese medicine for over two millennia, found worldwide application. Artemisinins are highly potent and safe anti-malarials, which are effective against multidrug-resistant *P. falciparum *[2-5].

One of the major goals identified to control malaria has been to prolong the lifespan of existing drugs by using drug-combination treatments. Artemisinin-based combination therapy (ACT) today includes artesunate-mefloquine, artemether-lumefantrine, artesunate-amodiaquine, artesunate-sulphadoxine-pyrimethamine and dihydroartemisinin-piperaquine [6]. ACT is currently recommended by WHO as the first-line treatment for uncomplicated malaria whereas recommendations for the treatment of severe malaria include artesunate or quinine given parenterally, followed by a course of an ACT [6]. Given the essential role of artemisinins in anti-malarial treatment, it is of great concern that resistance to artemisinins has recently emerged at the Thai-Cambodian border region [7-9]. While immediate action is necessary to conquer the spread of artemisinin resistance, the development of new tools to tackle malaria is even more urgent. The availability of the complete *P. falciparum *genome has facilitated identification of a series of novel candidate targets. This includes a large number of solute transport proteins that are underexploited as potential anti-malarial targets [10]. Here we describe recent advances in the development of the *P. falciparum *hexose transporter, PfHT, as a novel drug target.

### A novel approach to kill the malarial parasite - inhibition of sugar uptake

Blood is a steady and abundant source of glucose (~ 5 mM mean level) for malarial parasites residing and multiplying inside erythrocytes. Thus, it is not surprising that blood stages of malarial parasites are dependant on glucose as their main energy source. In line with this assumption, when malarial parasites are deprived of glucose, their intracellular ATP levels drop quickly along with their cytoplasmic pH [11]. Glucose deprivation also causes depolarization of the parasite plasma membrane [12]. The main source of ATP production in asexual blood stages of malarial parasites is glycolysis, which is followed by anaerobic fermentation of pyruvate to lactate. Although less efficient when compared with cellular respiration, glycolysis provides fast ATP production, which is required for the rapidly replicating intraerythrocytic parasite. The rate of ATP production by anaerobic glycolysis can be up to 100 times faster than that of oxidative phosphorylation. The role of the tricarboxylic acid (TCA) cycle in *Plasmodium *has long been a matter of debate. Recently, it has been discovered that, at least during their asexual blood stages, malarial parasites have atypical, branched TCA metabolism, which is largely disconnected from glycolysis and therefore plays a minor role in energy metabolism [13].

**Figure 1:**
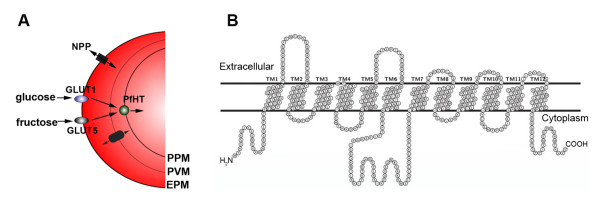
**Schematic representation of transport processes involved in uptake of hexoses in Plasmodium-infected erythrocytes (A) and predicted topology of PfHT (B). **EPM, erythrocyte plasma membrane; PVM, parasitophorous vacuole membrane; PPM, parasite plasma membrane; GLUT1, mammalian glucose transporter; GLUT5, mammalian fructose transporter; NPP, new permeability pathways (do not contribute significantly to the uptake of glucose [62]). B. Predicted topology of PfHT [18].

Glucose from blood is delivered to the intraerythocytic malarial parasite by sugar transporters present in the host and the parasite plasma membranes. Glucose is first transported from blood plasma into the erythrocyte cytosol by GLUT1, the facilitative glucose transporter highly abundant in the erythrocyte plasma membrane. Facilitative transporters, such as GLUT1, are passive carriers that move solutes down their concentration gradients [14]. As the malarial parasite resides inside the erythrocyte surrounded by an additional membrane, the parasitophorous vacuole (PV) membrane, glucose molecules must pass this membrane before reaching the parasite surface. The parasitophorous vacuole membrane is highly permeable to glucose and other solutes with molecular weights up to 1400 Da and these molecules pass freely between erythrocyte cytoplasm and the vacuolar space through high-capacity, low selectivity channels [15,16]. Finally, glucose uptake into the parasite is mediated by a facilitative transport process [17]. In the case of *P. falciparum*, PfHT (PlasmoDB accession number: PFB0210c) is the principal hexose transporter expressed in the parasite plasma membrane (Figure 1A) [18].

PfHT was first identified in the *P. falciparum *genome as a putative transporter gene showing homology to the major mammalian glucose transporter, GLUT1. Similar to GLUT1, the predicted topology of PfHT comprises 12 transmembrane helices with the amino and carboxy terminals located on the cytoplasmic side of the membrane (Figure 1B). Expression of PfHT in *Xenopus laevis *oocytes, enabled its detailed functional characterization. PfHT is a sodium-independent, saturable, facilitative hexose transporter [18]. While PfHT shares some of the typical sugar transporter features with the major mammalian glucose transporter, GLUT1, important mechanistic differences in their interaction with substrates have been identified. GLUT1 is selective for D-glucose (*K*_m _= 2.4 mM), whereas PfHT can transport both D-glucose (*K*_m _= 1 mM) and D-fructose (*K*_m _= 11.5 mM) (see Table 1). Fructose can replace glucose as an energy source when *P. falciparum *parasites are cultured *in vitro *[19]. Fructose gains entry to the red blood cell through the action of GLUT5, a facilitative fructose transporter present in the erythrocyte plasma membrane (Figure 1A) [20]. Uptake and metabolism of fructose probably do not play a significant physiological role for malarial parasite asexual blood stages, as plasma fructose concentrations are much lower than those of glucose, but may be important during parasite development inside the mosquito vector.

**Table 1:** Comparison of biochemical and kinetic properties of protozoan and mammalian hexose transporters

	Properties of hexose transport in parasites or host membranes	Characterization of hexose transporters genes in heterologous systems (*Xenopus *oocytes, CHO cells, Δ*lmgt*)		**Inhibition by compound 3361 K**_**i **_**(mM)**	Inhibition of glucose uptake by D-glucose analogues: important carbon position	Refs
				
	**K**_**m **_**for D-glucose or 2-DOG (mM)**	**K**_**m **_**for D-glucose or 2-DOG (mM)**	**Transport of D-fructose/K**_**m **_**(mM)**			
*P. falciparum* (PfHT)	~5 mM (6-DOG)	0.97 ± 0.34	+/11.6	0.053 ± 0.02	1,3,6	[18,30]

*P. knowlesi *(PkHT1)	ND	0.67 ± 0.16	+/5.12	0.108 ± 0.051	3,4,6	[30,63]

*P. vivax* (PvHT)	ND	0.52-0.85	+/5.91-19.03	0.112 ± 0.13	3,4,6	[30,63]

*P. yoelii* (PyHT1)	ND	0.12 ± 0.04	+/2.94	0.080 ± 0.016	3,4,6	[30,63]

*P. berghei* (PbHT)	ND	0.09 ± 0.02	0.54	~ 0.009	ND	[40]

*T. gondii *(TgGT1)	ND	0.030 ± 0.006	+ ^§^	2.073 ± 0.59	3,4,6	[63]

*B. bovis *(BboHT1)	ND	0.84 ± 0.50	+/ND	0.005 ± 0.002	4,6	[58]

*Leishmania *LmGT1	1.22 ± 0.22	ND	+/ND	ND	ND	[56]

*Leishmania *LmGT2	109 ± 22	ND	+/ND	ND	ND	[56]

*Leishmania *LmGT3	208 ± 40	ND	+/ND	ND	ND	[56]

*T. brucei* THT1	0.5-2.0	ND	+/2.56-3.91	ND	3,4	[55,64-66]

*T. brucei* THT2	0.038-0.08/2.0	0.151 ± 0.01	+/2.54	ND	3,4,6	[66]

*T. vivax* TvHT1	0.585 ± 0.068	0.545 ± 0.02	+/ND	ND	1,3,6	[65-67]

GLUT1	4-10	~ 2	-	3.3 ± 0.21	1,4,6	[19,66]

GLUT5	ND	-	+/13	ND	ND	[68]

ND - not determined

§ - D-fructose uptake at 37°C

+/ND - transport of fructose observed but K_m _not determined

Δ*lmgt - *glucose transporter null mutant of *Leishmania mexicana*

CHO - The Chinese hamster ovary (CHO) cell line

Mutational analyses of PfHT, together with studies using a variety of hexose analogues, were performed to investigate ligand-transporter interactions. Mechanistic differences that were observed between GLUT1 and PfHT in terms of their interaction with substrates, suggested that selective inhibition of PfHT may be possible. Several other features of PfHT favour it as a novel drug target. Most notably, *pfht *is a single copy gene without a close paralogue. The *P. falciparum *genome encodes three other proteins, PFI0955 w, PFI0785 c and PFE1455 w (PlasmoDB accession numbers) that have been annotated with putative sugar transport function, but they show considerably diverged amino acid sequences compared with that of PfHT (≤ 21% amino acid sequence identity) [21,22]. These putative transporters have not been investigated so far, thus their functions and cellular localizations remain unknown. Based on our genetic studies of PfHT (described later in this review), PFI0955 w, PFI0785 c or PFE1455 w do not function as alternative mechanisms for the delivery of hexoses as energy substrates for asexual blood stages of *P. falciparum*. Lack of functional redundancy is an important feature when potential drug targets are evaluated and prioritized. Also, a finding that there are no polymorphisms in PfHT amino acid sequence in eight laboratory and more than 20 field isolates analysed, adds value to PfHT as a drug target [23].

In addition to recognized therapeutic potential of PfHT, transporters that link glycolysis to apicoplast anabolism in malarial parasites may also become interesting as drug targets. The apicoplast is a relict plastid organelle that arose from a secondary endosymbiotic event [24]. This organelle is indispensable for parasite survival, which makes it attractive for therapeutic targeting. It contains four membranes and, therefore, requires complex transport systems. Despite being an anabolic organelle little is known about nutrient transport across its multiple membranes. Synthesis of fatty acids, isoprene precursors and haem is localized to the apicoplast and requires reduced carbon compounds and energy to be functional. The question of how these anabolic processes are fuelled in a non-photosynthetic plastid is of particular interest. Two transporters have been identified (PfiTPT (PFE1510 c) and PfoTPT (PFE0410 w)) that are localized in the innermost and outermost apicoplast membranes, respectively, and are homologous to plant transporters that move phosphorylated trioses, pentoses and hexoses across the chloroplast envelope in exchange for inorganic phosphate [25]. PfiTPT and PfoTPT have been proposed to act in tandem to transport glycolytic intermediates from the cytoplasm to the apicoplast, providing a source of carbon, reducing power and ATP required for biosynthetic processes [25]. Recently, using a cell-free transporter assay system, these two transporters have been found to show equivalent substrate preferences, with high substrate affinity for phosphoenolpyruvate and lower affinities for dihydroxyacetone phosphate and 3-phosphoglyceric acid, supporting the view that they act together to import these glycolytic intermediates into the apicoplast [26]. By being the link between cytoplasmic glycolysis and apicoplast anabolism, PfiTPT and PfoTPT are potential drug targets [25], but they require further validation steps.

### Genetic and chemical approaches to target validation

Validation of a particular parasite protein as a potential drug target can be performed by chemical or genetic tools. Chemical validation requires identification of a compound that specifically inhibits the parasite protein of interest without having an effect on the orthologous host proteins. Such a selective inhibitor also needs to demonstrate parasiticidal activity *in vitro *and/or *in vivo*. In certain cases, inhibition of parasite growth *in vitro *occurs at inhibitor concentrations lower than those necessary to block the transporter in a heterologous expression system. Various factors can account for this inhibitory difference between the two techniques, including the level of transporter expression, environmental factors that influence ligand binding (*e.g*. the composition of the lipid membrane) and inhibitor accumulation mechanisms.

Genetic validation involves a genetic modification of the parasite to "knock-out" a gene and show that loss-of-function of the protein results in non-viable or severely disabled parasites [27]. Also, if a specific inhibitor is available, the parasite can be genetically modified to over-express the potential target protein. In this case, demonstration of reduced inhibition with over-expression of the protein validates that protein as the molecular target of the applied drug.

Due to high target attrition rates during drug discovery projects, it is desirable that both chemical and genetic validation data are available for a novel target. Both of these methods have specific advantages but are also characterized with certain weaknesses. Chemical target validation provides information addressing the key druggability issues of cell permeability, selective toxicity and drug metabolism [28]. Also, it enables identification of non-protein targets. Genetic approaches, on the other hand, enable validation of targets with unknown function or for which highly specific inhibitors are not available. With the chemical validation approach, it is sometimes difficult to demonstrate the correlation between the target inhibition and the phenotype. Genetic validation, therefore, presents the "gold standard" for providing evidence for an essential function of a protein. Generally, the inability to obtain a chromosomal null mutant for a certain gene suggests the essential function of that gene for growth or survival but to definitely prove this, 'rescue' of the chromosomal null mutant must be achieved through expression of another copy of the gene. On the other hand, a gene knock-out that results in the growth of viable parasites suggests that the gene is not essential and probably not a drug target. It is important to note, however, that in certain circumstances it is possible to obtain viable organisms with the knock-out of an essential gene. This is possible through the occurrence of genetic changes at other loci that compensate for the lethal effect of the knock-out [28], or because *in vitro *conditions for growth are more permissive than those pertaining *in vivo*. This emphasizes the importance of both genetic and chemical validation approaches. Spontaneous amplification of an alternative transporter has, for example, been observed in a *Leishmania mexicana *glucose transporter null mutant (see below) [29]. Also, a gene that can be disrupted at one particular life cycle stage of a parasite may have an essential function during other life cycle stages.

### Chemical validation of PfHT as a drug target

**Figure 2:**
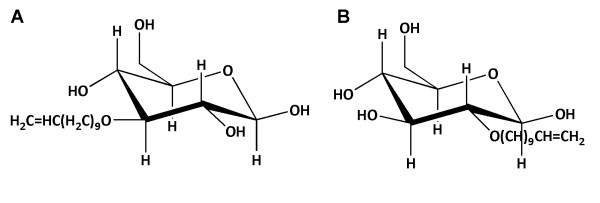
**Structures of the glucose-derived PfHT inhibitors**. A. 3-O-(undec-10-en)-1-yl-D-glucose (Compound 3361); B. 2-O-(undec-10-en)-1-yl-D-glucose.

Besides enabling functional studies, an additional value of the established robust heterologous expression system of PfHT in *X. laevis *oocytes was the opportunity to screen a series of glucose analogues and other compounds for their inhibitory properties. In an early study and based on previous findings with hexose analogues, a library of glucose derivatives with varying chain lengths at the C-3 position was generated and studied [30]. One of the compounds, 3-*O*-[undecyl-10-en]-1-yl-D-glucose, also known as compound 3361 (Figure 2A), was identified as an effective inhibitor of PfHT-mediated glucose transport in *Xenopus *oocytes as well as demonstrating a high degree of selectivity for PfHT over GLUT1 (see Table 1). The *K*_i _value of compound 3361 on glucose uptake by PfHT was found to be 53 μM. Compound 3361 killed *P. falciparum in vitro *(IC_50 _of 15 µM) and also showed activity in an *in vivo *murine model; a significant suppression of *Plasmodium berghei *parasitaemia was shown in a standard four-day suppression test [30]. These studies chemically validated PfHT as a novel drug target. Demonstration that compound 3361 acts via inhibition of transport rather than affecting the metabolism of glucose came from a study that measured uptake of non-metabolizable glucose analogues in *P. falciparum *parasites freed from erythrocytes by saponin lysis [31].

The therapeutic potential of PfHT has been further supported by findings that its orthologues in *Plasmodium vivax *and *Plasmodium knowlesi *are susceptible to inhibition by compound 3361 [32]. This compound also kills short-term cultures of *P. vivax *isolated from patients [32]. These findings suggest that if anti-malarials based on the inhibition of hexose transport are developed, they could be used for the treatment of non-falciparum malarias as well.

Later studies, using the *Xenopus *expression system of PfHT, further investigated inhibitory properties of sugar-based derivatives. A 3-*O*-substituted chain length of between 8-13 carbons is necessary to maintain inhibitory activity against PfHT [33]. Also, repositioning of the *O*-[undecyl-10-en]-1-yl substituent at C2 in the glucose molecule produced a significantly higher affinity inhibitor (K_i _of 2 μM against PfHT, see Figure 2B) compared with the C3 derivative, compound 3361 [34]. Similar to compound 3361, 2-*O*-[undecyl-10-en]-1-yl-D-glucose retained selective inhibitory activity against PfHT over GLUT1 and also inhibited *P. falciparum *growth *in vitro *[34].

PfHT can also be inhibited by compounds that do not have glucose-based structures. Recently, we have explored inhibitory potential of catechins, flavonoid compounds naturally found in green tea, against parasite and mammalian hexose transporters [35]. This study was driven by findings that some catechins inhibit mammalian GLUT1 and also show anti-malarial properties [36,37]. As with GLUT1, it was found that catechins containing a gallate group (epicatechin-gallate and epigallocatechin-gallate) inhibit PfHT and mammalian GLUT5. However, although these compounds inhibited hexose uptake processes in infected erythrocytes, the primary mechanism of their anti-malarial activity probably involves interaction with alternative higher affinity target/s [35].

### Genetic validation of essential function of PfHT for asexual blood stages

To add further support for PfHT as a novel drug target, *pfht *was investigated using genetic tools. Since the malarial parasite is haploid for a greater part of its life cycle (except for the short zygote and ookinete stages inside the mosquito midgut), reverse genetic studies of an essential gene are not straightforward, as the knock-out of such a gene would have a lethal effect. Therefore, in genetic studies of *pfht*, a knock-out/complementation system was used, which has been successfully used in research of *P. falciparum *kinases [38]. With this approach, a knock-out of a gene of interest is attempted with or without the presence of a complementation vector that allows episomal, "rescue", expression of the gene of interest. If the gene is essential, its knock-out would be lethal for the parasite and will only be achieved in the presence of a rescuing, complementation vector. In this way, it was established that *pfht *is essential for survival of asexual blood stages [39]. Additionally, a transgenic *P. falciparum *line overexpressing *pfht *is less susceptible to growth inhibition by compound 3361 compared with wild-type parasites. This transgenic line may represent a useful tool to link activities of future putative PfHT inhibitors to the target. The essential function of the hexose transporter for survival of blood stages was confirmed in a rodent malarial model, *P. berghei*. The *P. berghei *hexose transporter gene - *pbht *(PlasmoDB accession number: PB000562.01.0), is accessible for genetic targeting, as shown by *gfp*-tagging of the gene, but is refractory to knock-out attempts [39]. Recently, this finding has been reproduced, using a similar genetic approach [40].

Therefore, PfHT has been validated as a drug target both chemically and genetically, and so far it is the only transport protein for which both validation approaches have been undertaken.

### Life cycle studies

**Figure 3:**
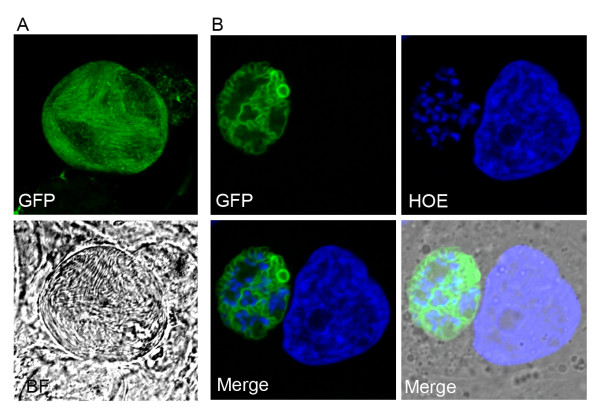
**Direct fluorescence imaging of transgenic *Plasmodium berghei *parasites expressing *Plasmodium berghei *hexose transporter (PbHT) fused to a GFP protein**. a) an oocyst formed inside a mosquito midgut (21 days post infection) containing several hundreds of newly formed sporozoites [39]; b) exoerythocytic schizont formed inside a human hepatoma (Huh-7) cell 67 hours post-infection [41]. Note that the pattern of staining is consistent with localization of PbHT to the parasite plasma membrane, which invaginates around clusters of nuclei during the formation of merozoites. GFP, *pbht-gfp P. berghei*; HOE, Hoechst 33342 (used as a nuclear dye); BF, bright-field image.

In addition to an essential function of PfHT for asexual blood stages of the malarial parasite, this transporter is also important for parasite development and/or survival at other stages of its complex life cycle. This has been investigated using transgenic *P. berghei *lines, amenable to *in vivo *life cycle studies. PbHT expression was analysed by direct imaging of live parasites expressing PbHT fused to a GFP reporter [39,41] and by immunofluorescence analysis of a transgenic line expressing PbHT fused to the haemagglutinin epitope [40]. PbHT was constitutively expressed throughout the parasite's development inside the mosquito vector as well as during the hepatic stages (see Figure 3 for examples). This is in keeping with previous transcriptomic and proteomic studies [42-46].

Glucose transport by plasmodial hexose transporters is necessary for normal development of insect stages (microgametes, ookinetes and oocysts), as shown by inhibitory studies with compound 3361. Compound 3361 reduced the number of oocysts formed inside infected mosquito midguts, when tested using a membrane feed assay [41] as well as when mosquitoes were fed on compound 3361 treated, *P. berghei*-infected mice [40]. Compound 3361 also affects exflagellation of male gametes and ookinete production in a dose-dependant manner with IC_50 _values of between 100 to 300 μM [40,41], although only the former in a glucose-sensitive manner [41]. PfHT targeting thus holds some potential for transmission blocking strategies. The only current drugs that can kill *P. falciparum *gametocytes and, thus, reduce transmission are 8-aminoquinolines, such as primaquine [47]. The use of primaquine is hampered by the haemolysis this drug causes in patients with glucose-6-phosphate dehydrogenase (G6PD) deficiency [48]. Artemisinins inhibit early stages of gametocyte development.

Liver stages of the malarial parasite are also killed by compound 3361 (with an IC_50 _of 11 μM) [40,41]. This effect was not due to reduced viability of the host hepatoma cells or to inhibition of host glucose uptake pathways [41]. These data suggest that novel chemotherapeutic interventions that target PfHT may well be active against liver stages. Efficacy against malarial parasite liver stages has gained increasing attention as a desirable property of new anti-malarials [49]. Drugs that act at this stage, preventing sporozoite infection or replication of parasites in the liver, stop the infection from reaching the symptomatic blood stages. Since very limited number of drugs have this causal prophylactic activity (namely primaquine, proguanil and atovaquone), it is important to identify novel potential strategies.

### High throughput screening systems

After a target has been robustly validated, the next step of its development as a drug target requires a high throughput system for the screening of compound libraries. A promising system for functional expression of parasite and human glucose transporters has recently been developed based on a glucose transporter null mutant of *Leishmania mexicana*, named Δ*lmgt *[50,51]. *PfHT*, *T. brucei *hexose transporter *THT1 *and human *GLUT1 *have been successfully expressed in Δ*lmgt *heterologous system. It was demonstrated that functional complementation of Δ*lmgt *with parasite and human transporters can be used to identify inhibitory compounds specific for parasite glucose transporters [50]. An advantage of Δ*lmgt *as an expression system compared with mammalian cells is the absence of background glucose uptake. This background, characteristic for mammalian cells due to the presence of multiple endogenous glucose transporters, may affect the inhibition data obtained during experiments. The Δ*lmgt *system is amenable to high throughput screening by using a cell-permeable dye, alamarBlue™, which is reduced by viable parasites and in its reduced form emits a strong fluorescence signal that is a quantitative measure of the parasite count [50]. Recent expression of PfHT in a yeast mutant cell line deficient in endogenous hexose transporters may potentially be useful in high-throughput studies of inhibitors [40], although this system requires further validation, including comparisons to yeast mutants expressing mammalian glucose transporters as well as development of a screening assay methodology.

Several thousand new chemical entities with *in vitro *activity against both sensitive and multidrug resistant *P. falciparum *were discovered recently [52,53]. These newly identified libraries of active compounds may serve as an excellent starting point for target-based lead discovery, which has not produced satisfactory results so far, mostly due to lack of whole-cell activity [52].

### Potential of glucose transporters of other pathogens as drug targets

In addition to malarial parasites, the uptake of glucose is essential for the viability of several other protozoan pathogens. This has been established for *Trypanosoma brucei*, a pathogen causing sleeping sickness. Sleeping sickness is fatal if untreated and novel trypanocides are required because current treatments are not satisfactory due to a lack of efficacy, poor safety profiles, difficulties in administration and/or high cost [54]. Glycolysis has been considered a promising target for new drugs against trypanosomiasis. The reason for this, in addition to glycolysis being essential for the survival of blood stage parasites, is the unique nature of this pathway in *Trypanosoma *[54]. The first seven reactions of the glycolytic pathway happen inside peroxisome-like organelles called glycosomes and the enzymes involved display unique structural and kinetic properties, which are distinct from those of the mammalian host. Glucose uptake in *T. brucei *happens via two glucose transporters, THT1 and THT2 (*Trypanosoma *hexose transporter 1 and 2) (Table 1). The lower glucose affinity THT1 (K_m _~1 mM), is the isoform expressed in the blood stages, whereas the high-affinity THT2 (K_m _~0.05 mM) is expressed during insect stages. Glucose and fructose analogues can inhibit glucose uptake by THT1 and kill the blood forms of *T. brucei *[55]. THT1, therefore, may represent a good drug target and its heterologous expression, which has been achieved in the *L. mexicana *glucose transporter null mutant mentioned before, may facilitate the search for a specific inhibitory compound [50].

Glucose is also an essential nutrient for the infectious stages of *L. mexicana*, an intracellular parasite of macrophages, which causes cutaneous leishmaniasis. The uptake of glucose is essential for proliferation of disease-causing amastigote stages of *L. mexicana *[56]. Glucose uptake in *L. mexicana *is mediated by three glucose transporter isoforms, LmGT1, LmGT2 and LmGT3 (*Leishmania mexicana *glucose transporter 1, 2 and 3) (Table 1) [56]. The three glucose transporter isoforms are very similar in their protein sequences but they have specific subcellular localizations and display developmentally-regulated expression [56,57]. The importance of hexose transport for infectious stages of *L. mexicana *has been further supported by a recent discovery of spontaneous suppressors of the glucose transporter null mutant, Δ*lmgt *[29]. These suppressor mutants have regained the ability to transport hexoses through an amplification of an alternative glucose transporter gene, the LmGT4 permease, and have also partially restored virulence [29]. Thus, although inhibition of *L. mexicana *glucose transporters has not been investigated (in the way it has been done for *P. falciparum *and *T. brucei *hexose transporters, using glucose analogue-based inhibitors), interference with their function may show therapeutic potential.

*Babesia *are tick-borne haemoprotozoan apicomlexan parasites, sharing many similarities, including their asexual blood-stage proliferation with *Plasmodium*. *Babesia bovis *causes severe infections in cattle and imposes a high economic burden. *Babesia bovis *hexose transporter 1 - BboHT1 (Table 1), one of the two hexose transporters identified in the genome of this parasite, has been expressed and characterized in *Xenopus *oocytes [58]. BboHT1 is approximately 10-fold more sensitive to inhibition by compound 3361 compared with PfHT but this compound does not affect parasite growth [58]. It is possible that compound 3361 may not reach the intraerythrocytic site of BboHT1 or that another D-glucose transport pathway, insensitive to compound 3361, is present [58]. Alternatively, the parasite may not rely completely on glucose as an energy source. The authors have suggested that the parasites may oxidize glutamate via glutamate dehydrogenase, which is present in blood stages, and feed the resulting α-ketoglutarate into the tricarboxylic acid cycle [58]. Evidence obtained so far, therefore, cannot support BboHT1 as a drug target.

*Toxoplasma gondii*, an obligate intracellular parasite of the *Apicomplexa *phylum related to *Plasmodium*, can tolerate the deletion of its surface sugar transporter without an effect on its virulence [59]. The authors of this study have suggested that in *T. gondii *glucose metabolism is dispensable because of glutaminolysis. The ability to use alternative energy sources has probably co-evolved with the ability of these parasites to replicate in most cell types. Thus, phylogenetically closely related parasites, such as *Toxoplasma, Babesia *and *Plasmodium*, can fundamentally differ in terms of their dependence on glucose and essentiality of their glucose transporters. During their life cycles, the ability of some parasites to use alternative energy sources may depend on the specific life cycle stage. For example, insect stages of *T. brucei *(procyclic forms) and *L. mexicana *(promastigotes) can thrive in glucose-depleted medium [60,61], whereas the growth of their mammalian stages is dependant on glucose (as described above).

## Conclusions

**Figure 4:**
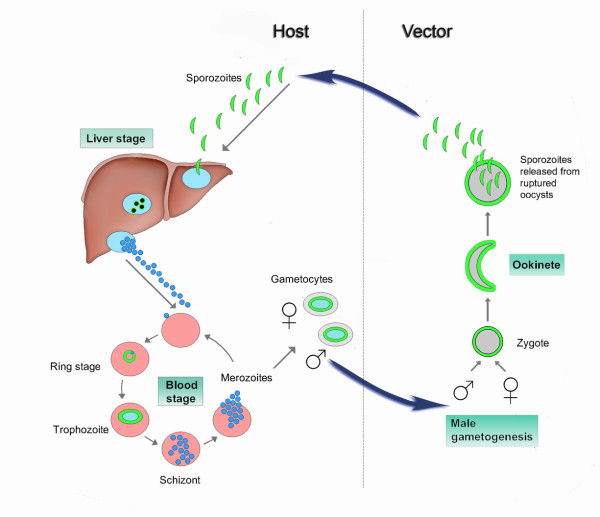
**Schematic representation of the life cycle of *Plasmodium *parasites showing a summary of inhibition and expression studies of plasmodial hexose transporters**. Parasites shown in green represent a *pbht-gfp *transgenic line, which has been analysed via direct fluorescence imaging. Using this transgenic line, expression of PbHT-GFP was observed at 24, 48 and 67 h post infection in the liver stage [41], in both early and late asexual blood stages, in sexual blood stage forms (gametocytes) and in female gametes/or zygotes, ookinetes, midgut oocysts and sporozoites derived from mosquito midgut oocysts and salivary glands [39]. Highlighted life cycle stages (blue boxes) show where compound 3361 inhibits parasite growth and development; in the liver stages *in vitro*, *P. berghei *parasite growth is inhibited by compound 3361 with an IC_50 _value of 11 μM [41]; in the asexual blood stages *in vitro*, *P. falciparum *parasite growth is inhibited by compound 3361 with an IC_50 _value of 16 μM and *in vivo *P. *berghei *parasite growth is reduced by 40% (4-day suppression test; 25 mg/kg; administered i.p. twice daily) [30]; in *P. berghei *sexual stages male gametogenesis (IC_50 _value of 286 μM) and ookinete production (IC_50 _value of 252 μM) are inhibited by compound 3361 [41].

Hexose transporters of the three most pathogenic malarial parasites, *P. falciparum*, *P. vivax *and *P. knowlesi*, have been validated as novel drug targets. In addition to an essential role of plasmodial hexose transporters during parasite blood stages, these transporters may also have a crucial role for liver and insect stages of malarial parasite life cycles (see Figure 4 for summary).

Glucose transport has also been found to be essential for the viability of *T. brucei *and *L. mexicana *infectious stages. Thus, inhibition of hexose transport may present an attractive approach in the development of other new anti-parasitic drugs, in addition to the discovery of novel anti-malarials.

## Abbreviations

BboHT1: *Babesia bovis *hexose transporter 1; GLUT1/5: mammalian facilitative glucose transporter 1/5; LmGT1/3: *Leishmania mexicana *glucose transporter 1/3; PfHT: *Plasmodium falciparum *hexose transporter; THT1/2: *Trypanosoma brucei *hexose transporter 1/2.

## Competing interests

The authors declare that they have no competing interests.

## Authors' contributions

All authors contributed in the conception of the article; KS and HMS drafted the manuscript; ETD generated table, figures (1 and 4) and provided text for subsections. HMS and SK critically reviewed and revised the manuscript. All authors read and approved the final manuscript.
